# Critical Care Transport: Blunt Polytrauma in Pregnancy

**DOI:** 10.21980/J81366

**Published:** 2025-07-31

**Authors:** Emma Rolf, Samuel Kefer, Jennifer Quinn, Ryan Newberry, Andrew Cathers, Craig Tschautscher, Brittney Bernardoni

**Affiliations:** * University of Wisconsin, Madison, WI; ^ University of Wisconsin, Department of Emergency Medicine, Madison, WI; † University of Wisconsin Health, Med Flight, Madison, WI

## Abstract

**Audience:**

This simulation is designed for critical care transport nurses and attending physicians. It can also be adapted for critical care transport paramedics and respiratory therapists as well as emergency medicine nurses, residents, and attending physicians.

**Introduction:**

Emergency and trauma surgery practitioners routinely perform primary and secondary surveys as a systematic approach to trauma care. While this approach has broad applications, clinicians must also be versed in the nuances of caring for special populations in trauma. One such example is the obstetric patient. The incidence of trauma in pregnancy is increasing and is now the leading cause of non-obstetrical maternal death in the United States.^1^ Optimal maternal resuscitation depends on an understanding of the significant anatomic and physiologic changes of pregnancy and their influence on airway, breathing, and circulation.^2^,^3^,^4^

This case presents a blunt polytrauma with unstable pelvic and lower extremity fractures precipitating hemorrhagic shock and the need for blood product transfusion. Learners must quickly adapt their clinical acumen and consider the influence of an obviously gravid patient on their resuscitation. Implementing and practicing the required skills allows for delivery of high-quality care. This session ensures that learners have a well-rounded understanding of scenarios that could occur in the resuscitation of a pregnant trauma patient.

**Educational Objectives:**

At the completion of this simulation participants will be able to 1) perform primary and secondary trauma surveys, 2) assess the neurovascular status of a tibia/fibula fracture, 3) appreciate anatomic and physiologic differences in pregnancy, 4) appropriately order analgesia and imaging, 5) recognize and treat hemorrhagic shock, 6) perform an extended focused assessment with sonography in trauma exam (eFAST) in undifferentiated hemorrhage, 7) identify a displaced pelvic fracture and properly apply a pelvic binder, and 8) obtain and interpret fetal heart rate using ultrasound.

**Educational Methods:**

This is a high-fidelity simulation portraying a 24-year-old pregnant female who requires hemodynamic resuscitation, pelvic and extremity fracture stabilization, and assessment of fetal heart rate. After completion of the simulation, learners will participate in a debrief and small group discussion that focuses on didactic knowledge and its application to patient care, crew resource management, and interprofessional communication.

**Research Methods:**

Learners were required to complete a pre- and post-simulation test evaluating their knowledge of pregnant trauma patient care. The results were then compared to evaluate whether the simulation improved participants’ knowledge base. Learners also completed an evaluation of the simulation case itself using a 5-point Likert scale and free response. Feedback from the first round of simulations was used to modify the simulation case prior to the second round.

**Results:**

Our simulation included 26 participants: nine attending emergency medicine/critical care transport physicians and 17 critical care transport nurses. All participants took a pre- and post-test evaluating their medical knowledge with an average score of 60% and 93.4% correct responses respectively. In addition, participants were given the opportunity to evaluate the simulation itself via an anonymous survey. All (100%) of the participants strongly agreed that the content was relevant, met educational needs, was effective, and was appropriate for professional licensure level.

**Discussion:**

This simulation, focusing on the care of a pregnant trauma patient, was well received by the learners and effectively met educational goals at an appropriate level for professional licensure. Participants demonstrated an excellent understanding of appropriate imaging evaluation/interpretation, blood product resuscitation, and use of tranexamic acid (TXA) in the pregnant trauma patient. Improvement in interpretation of fetal heart rate as well as use/application of a pelvic binder in the setting of pregnancy were seen as a result of this simulation training.

**Topics:**

Pregnant trauma, fetal heart rate, pelvic fracture, blood product transfusion, extremity fracture, critical care transport, emergency medicine simulation.

## USER GUIDE

**Table:** 

List of Resources:	
Abstract	1
User Guide	3
Instructor Materials	5
Operator Materials	13
Debriefing and Evaluation Pearls	16
Simulation Assessment	20

**Learner Audience:**

Medical Students, Interns, Junior EM Residents, Senior EM Residents, Attending Physicians, Nursing and Ancillary Staff

**Time Required for Implementation:**

**Instructor Preparation:** 20 minutes

**Time for case:** 15 minutes

**Time for debriefing:** 20 minutes

**Recommended Number of Learners per Instructor:**

2–5 learners/instructor

**Topics:**

*In situ* simulation, simulation competition, LVAD, left ventricular assist device.

**Objectives:**

By the end of this simulation, learners will be able to:

1. Perform a primary and secondary trauma survey
2. Assess the neurovascular status of a tibia/fibula fracture
3. Appreciate anatomic and physiologic differences in pregnancy
  - SaO2 goal > 94%; EtCO2 goal 30
  - Transport in left lateral tilt position (maximize fetal/maternal perfusion)
4. Appropriately order analgesia and imaging
5. Recognize and treat hemorrhagic shock
  - Manually displace uterus leftwards to decrease uterocaval compression
  - Start fresh frozen plasma/packed red blood cells
  - Administer TXA/Ca
6. Perform eFAST
7. Identify pelvic fracture and stabilize with pelvic binder
8. Use point of care ultrasound to assess fetal heart rate

### Linked objectives and methods

Blunt polytrauma is a common presentation for critical care transport and trauma care practitioners alike. This case requires learners to apply the fundamentals of trauma care by performing a primary and secondary survey (objective 1). Obvious deformity of the left lower extremity should prompt assessment of neurovascular status (objective 2). Learners should apply their knowledge of anatomic and physiologic adaptations in pregnancy that can optimize maternal status by targeting a goal peripheral arterial oxygen saturation (SpO2) of greater than 94%, goal end-tidal carbon dioxide levels (EtCO2) of 30mmHg, and consideration of transport in left lateral tilt position to minimize uterocaval compression (objective 3). Analgesia and appropriate imaging should be ordered (objective 4). Synthesis of initial assessment of pale appearance, tachycardia, and hypotension should lead to recognition of hemorrhagic shock (objective 5) and prompt manual displacement of the uterus, blood product resuscitation with fresh frozen plasma (FFP) and packed red blood cells (PRBC), and the administration of tranexamic acid (TXA) and calcium (objectives 5a, 5b, 5c). With evidence of hemorrhagic shock on exam, an eFAST should be performed (objective 6). Pelvis X-ray returns and shows displaced fracture that should prompt learners to apply a pelvic binder (objective 7). After stabilization, fetal heart rate should be obtained using point of care ultrasound (objective 8). Patient is transported to the receiving facility.

### Recommended pre-reading for instructor

- Barraco RD, Chiu W, Clancy T, et.al. Practice management guidelines for the diagnosis and management of injury in the pregnant patient: The EAST Practice Management Guidelines Work Group. *J Trauma*. 2010;69: 211–214.
- Rizzo A, Martin M, Inaba K, et.al. Pregnancy in trauma—A Western Trauma Association algorithm. *J Trauma Acute Care Surg* 93(4):p e139–e142, October 2022.
- Downing J, Sjeklocha L. Trauma in pregnancy. *Emerg Med Clin North Am.* 2023; 41(2):223–245.
- Amal Mattu. *EMCast: June 2023 (2) Trauma in Pregnancy*. June 12, 2023. Accessed 05/15/24. https://podcasts.apple.com/us/podcast/emedhome-com-emcast/id1481103144?i=1000616658805

### Results and tips for successful implementation

This is a high-fidelity simulation portraying a 24-year-old pregnant female who requires hemodynamic resuscitation, pelvic and extremity fracture stabilization, and assessment of fetal heart rate. By following the simulation events table, participants will understand the importance of interprofessional collaboration to stabilize a critically ill patient.

This simulation case was run eight times divided between two sessions two weeks apart in December of 2023 as part of a bi-monthly critical care transport simulation education. Twenty-six learners participated, including nine attending emergency medicine/critical care transport physicians and 17 critical care transport nurses. Groups were composed of one physician and one nurse performing the simulation (mirroring the composition of our transport team), with an additional nurse functioning as an embedded actor playing the role of the bedside nurse from the sending hospital. Facilitators included one physician and one nurse who together presented the case, ran the mannequin/vitals, and debriefed the participants.

Participants’ medical knowledge was assessed via a pre and post-test which demonstrated improvement in average score from 60% pre- to 93.4% post-simulation participation. The largest improvement was seen on questions assessing evaluation of extremity fracture and interpretation of fetal heart rate. In addition, participants were given the opportunity to evaluate the simulation itself via an anonymous survey. All (100%) of participants strongly agreed (highest rating possible) that the content was relevant, effective, appropriate for professional licensure level, and met educational needs. Learners specifically commented on the relevant complexity of the case and appropriate tasks for each participant role. Facilitators also provided feedback on the simulation case and debriefing session. Both participant and facilitator feedback from the first session was used to modify the case before the second session. Specifically, confederate prompting to assess extremity fracture and mannequin prompting to assess fetal heart rate were added to the second session. The case presented here represents the finalized version incorporating both learner and facilitator feedback from both sessions.

## Supplementary Information



## Figures and Tables

**Table:** 

#1	Pelvic Radiograph
#2	Chest Radiograph (CXR)
#3	Tib/Fib Radiograph

**Table:** 

**Vitals:**	Heart Rate (HR) 97	Blood Pressure (BP) 105/77
	Respiratory Rate (RR) 24	Temperature (T) 98.7°C
	Oxygen Saturation (O_2_Sat) 93%	

**Figure:**
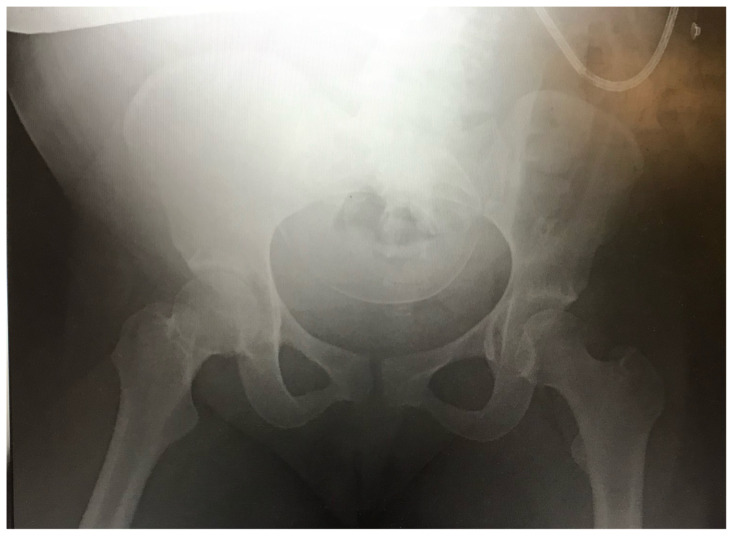


**Figure:**
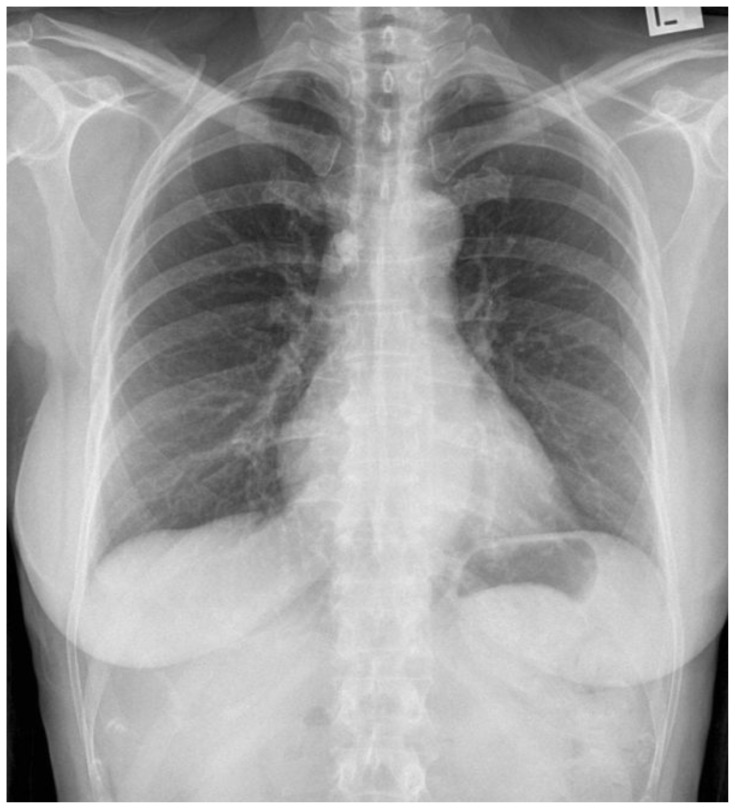


**Figure:**
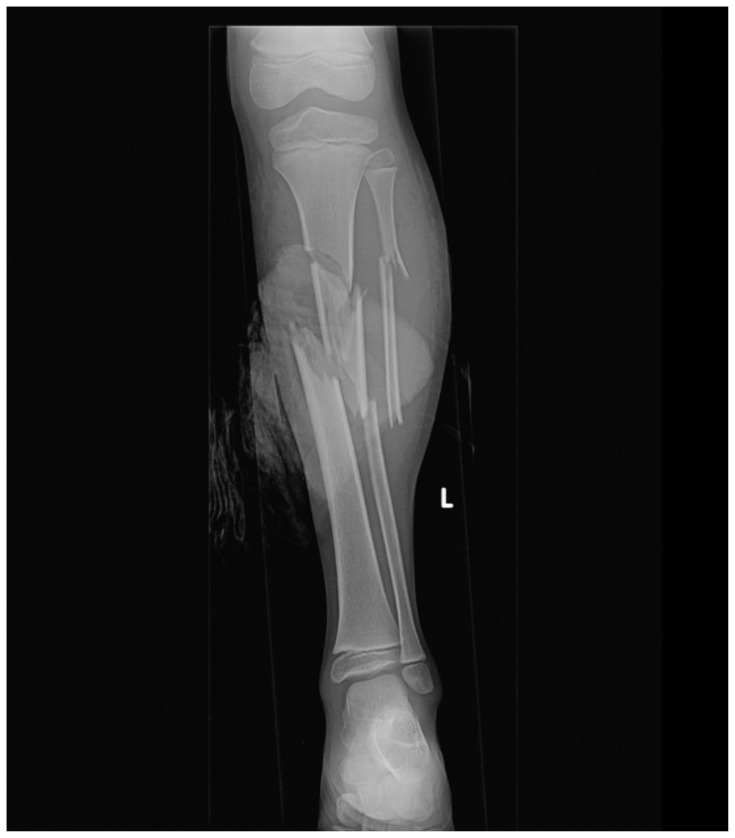


**Table:** SIMULATION EVENTS TABLE:

Minute (state)	Participant action/trigger	Patient status (simulator response) & operator prompts	Monitor display (vital signs)
0:00 (Baseline)	Perform primary survey Obtain vital signs Establish IV access Start second IV	Patient will be lying on stretcher yelling in pain.	T: 98.7º C HR: 97 BP: 100/77 RR: 24 O2: 93% on room air
1:00	Perform secondary survey Recognize pregnancy Apply supplemental O2	If no supplemental oxygen applied, nurse asks, “Do pregnant patients have any different goals for vital signs?” If no supplemental oxygen applied after prompt, oxygen saturation drops mildly.	T: 98.7º C HR: 97 BP: 100/77 RR: 24 O2: 100% on 2L nasal cannula (NC) O2: 88% if no intervention
3:00	Call for X-ray May give analgesia	If no X-ray ordered, nurse prompts, “Do you need me to call for any imaging?” If no analgesia given, nurse asks, “Is there anything we can give her for pain?”	Unchanged
5:00	Recognize hemorrhagic shock Call for blood products and start balanced transfusion May give TXA and calcium	If no intervention, nurse says, “Our blood pressure is dropping; what should we do?” If no blood/crystalloid given or if vasopressors started, blood pressure continues to drop and patient becomes more tachycardic.	HR: 110 HR: 127 if no intervention BP: 88/57 BP: 80/47 if no intervention RR: 20 SPO2: 100% on 2L NC
7:00	Perform eFAST Return of CXR and pelvis x-ray Diagnose pelvic fracture, apply binder	If no intervention, nurse asks, “Where do you think the blood could be going?” If no response “Do you want me to grab an ultrasound?” If failure to identify pelvic fracture call from radiology. “I am worried she has an open book pelvic fracture.” If no intervention, nurse says, “We have a pelvic binder bedside if you would like to place one.”	HR: 110 BP: 90/60 RR: 20 SPO2: 100% on 2L NC
9:00	Continue transfusion Manually displace uterus position in left lateral tilt	If hypotension not noted, nurse says, “The blood pressure isn’t coming up yet; what else can we do?” If blood not given, patient becomes more hypotensive and tachycardic. If not repositioned, nurse says, “We have a wedge or pillows if you think rolling may help.”	HR: 110 HR: 126 if no intervention BP: 88/60 BP: 80/49 if no intervention RR: 20 SPO2: 100% on 2L NC
11:00	Obtain fetal heart rate after stabilization	Mom asks, “Is my baby, okay?” If no intervention, nurse asks, “Would you like me to find a fetal doppler?”	HR: 95 BP: 110/70 RR: 20 SPO2: 100% on 2L NC
13:00	Discuss transfer with receiving trauma center Case completion	If no discussion of transfer, nurse asks, “Do you want me to get the access center on the line?”	Unchanged from above

**Table:** 

**Critical Actions:** Perform primary survey with recognition of hypotension and neurovascular assessment of extremity with obvious fracture Connect patient to cardiopulmonary monitors and establish two points of large bore IV access Identify gravid abdomen on secondary survey and recognize the impacts of maternal physiology Recognize presentation of hemorrhagic shock and start balanced blood product resuscitation considering adjuncts of TXA and calcium administration Identify displaced pelvic fracture and apply pelvic binder Relieve uterocaval compression to optimize maternal hemodynamics Obtain fetal heart tones after stabilization Arrange transport to receiving trauma center	0:00

**Table:** 

	Milestone	Did not achieve level 1	Level 1	Level 2	Level 3
1	**Emergency Stabilization (PC1)**	□ Did not achieve Level 1	□ Recognizes abnormal vital signs	□ Recognizes an unstable patient, requiring intervention Performs primary assessment Discerns data to formulate a diagnostic impression/plan	□ Manages and prioritizes critical actions in a critically ill patient Reassesses after implementing a stabilizing intervention
2	**Performance of focused history and physical (PC2)**	□ Did not achieve Level 1	□ Performs a reliable, comprehensive history and physical exam	□ Performs and communicates a focused history and physical exam based on chief complaint and urgent issues	□ Prioritizes essential components of history and physical exam given dynamic circumstances
3	**Diagnostic studies (PC3)**	□ Did not achieve Level 1	□ Determines the necessity of diagnostic studies	□ Orders appropriate diagnostic studies. Performs appropriate bedside diagnostic studies/procedures	□ Prioritizes essential testing Interprets results of diagnostic studies Reviews risks, benefits, contraindications, and alternatives to a diagnostic study or procedure
4	**Diagnosis (PC4)**	□ Did not achieve Level 1	□ Considers a list of potential diagnoses	□ Considers an appropriate list of potential diagnosis May or may not make correct diagnosis	□ Makes the appropriate diagnosis Considers other potential diagnoses, avoiding premature closure
5	**Pharmacotherapy (PC5)**	□ Did not achieve Level 1	□ Asks patient for drug allergies	□ Selects an medication for therapeutic intervention, consider potential adverse effects	□ Selects the most appropriate medication and understands mechanism of action, effect, and potential side effects Considers and recognizes drug-drug interactions
6	**Observation and reassessment (PC6)**	□ Did not achieve Level 1	□ Reevaluates patient at least one time during case	□ Reevaluates patient after most therapeutic interventions	□ Consistently evaluates the effectiveness of therapies at appropriate intervals
7	**Disposition (PC7)**	□ Did not achieve Level 1	□ Appropriately selects whether to admit or discharge the patient	□ Appropriately selects whether to admit or discharge Involves the expertise of some of the appropriate specialists	□ Educates the patient appropriately about their disposition Assigns patient to an appropriate level of care (ICU/Tele/Floor) Involves expertise of all appropriate specialists
9	**General Approach to Procedures (PC9)**	□ Did not achieve Level 1	□ Identifies pertinent anatomy and physiology for a procedure Uses appropriate Universal Precautions	□ Obtains informed consent Knows indications, contraindications, anatomic landmarks, equipment, anesthetic and procedural technique, and potential complications for common ED procedures	□ Determines a back-up strategy if initial attempts are unsuccessful Correctly interprets results of diagnostic procedure
20	**Professional Values (PROF1)**	□ Did not achieve Level 1	□ Demonstrates caring, honest behavior	□ Exhibits compassion, respect, sensitivity and responsiveness	□ Develops alternative care plans when patients’ personal beliefs and decisions preclude standard care
22	**Patient centered communication (ICS1)**	□ Did not achieve level 1	□ Establishes rapport and demonstrates empathy to patient (and family) Listens effectively	□ Elicits patient’s reason for seeking health care	□ Manages patient expectations in a manner that minimizes potential for stress, conflict, and misunderstanding. Effectively communicates with vulnerable populations, (at risk patients and families)
23	**Team management (ICS2)**	□ Did not achieve level 1	□ Recognizes other members of the patient care team during case (nurse, techs)	□ Communicates pertinent information to other healthcare colleagues	□ Communicates a clear, succinct, and appropriate handoff with specialists and other colleagues Communicates effectively with ancillary staff

## INSTRUCTOR MATERIALS

**Case Title:** Critical Care Transport: Blunt Polytrauma in Pregnancy

**Case Description & Diagnosis (short synopsis):** This scenario presents a 24-year-old obviously gravid female who has fallen down a flight of stairs and was brought by EMS to an outside ED. The critical care transport team arrives to conduct a trauma evaluation and stabilize for transfer. Primary survey shows an ill-appearing patient with an elevated shock index concerning for hemorrhagic shock. Secondary survey notes right chest wall bruising, pelvic pain with stable pelvis, and left tib/fib fracture. Fetal heart rate is assessed with point of care ultrasound. Providers should resuscitate by relieving uterocaval compression and starting blood product transfusion, with TXA and calcium administration as adjuncts. Initial imaging will return (CXR, pelvic XR, L Tib/Fib XR) and show a displaced pelvic fracture and tib/fib fracture. Providers will need to apply a pelvic binder and provide the patient with another transfusion in order to stabilize vital signs. Participants will then need to discuss a transport plan and package the patient for flight.

**Equipment or Props Needed:**

- High fidelity mannequin/simulator
  ○ Padded gauze over left tib/fib
  ○ Bruising over right chest wall
  ○ Pregnant
- Crash Cart
- Trauma Cart
  ○ Pelvic binder
  ○ Backboard
  ○ C-collar
- Gurney
- IV Pole

**Embedded actors needed:**

- One embedded actor to play the sending hospital bedside nurse.

**Stimulus Inventory:**

**Background and brief information:** This scenario takes place in a rural emergency department. The patient is a 24-year-old gravid woman who fell down a flight of stairs.

**Initial presentation:** The patient was brought in to the emergency department by ambulance after a mechanical fall down one flight of stairs. She has just been transferred from the emergency medical service’s (EMS) gurney to your stretcher and is awaiting initial evaluation.

**How the scene unfolds:** The patient is a 24-year-old woman G1P0 at 30 weeks gestation brought in by EMS after a mechanical fall down one flight of stairs. If prompted by learners, EMS is available to provide the following information: the patient’s husband witnessed her trip and fall down 13 steps. There was head strike, but no reported loss of consciousness. They have identified an obvious deformity to the left lower extremity. They are a basic EMS unit, and have not provided any interventions en route.

The patient is lying supine on stretcher with backboard and cervical collar. Learners should immediately start their primary survey, place patient onto cardiopulmonary monitors, obtain a full set of vital signs, and delegate nursing to establish two points of large bore IV access. The airway is patent, with patient crying out in pain. Patient is tachypneic, but without accessory muscle use, no critical hypoxemia, and bilateral breath sounds present. Circulation shows soft blood pressure, pale appearance, elevated shock index. There are palpable pulses present in all extremities. Glasgow Coma Score would be 15 with alert patient yelling in pain. Exposure reveals obvious deformity to the left lower extremity with concern for open fracture; the site does appear hemostatic. Secondary survey is notable for bruising over right chest wall and an obviously gravid abdomen, with uterus palpable midway between the xiphoid process and umbilicus.

Recognition of pregnancy should prompt learners to place patient on supplemental oxygen for SPO2 goal >94% and consider uterocaval displacement to optimize venous return. Learners should call for chest x-ray, pelvis x-ray, and x-ray of the left lower extremity. Analgesia should be given with obvious fracture on exam, and patient continuing to endorse pain.

Repeat vital signs will show increasing tachycardia and down trending blood pressure. The synthesis of patient’s history, exam, and vitals, should prompt diagnosis of hemorrhagic shock. Learners should initiate balanced blood product resuscitation with adjuncts of TXA and calcium administration. Undifferentiated source of hemorrhage should prompt use of ultrasound and eFAST exam. No free fluid or pneumothorax seen on eFAST. Chest X-ray shows no obvious rib fractures, no pneumothorax or hemothorax. Pelvis X-ray will return and show displaced pelvic fracture. Identification should prompt learners to place pelvic binder. Blood pressure does not improve with pelvic binder or blood product administration unless uterocaval compression is relieved with manual displacement or tilt to left lateral decubitus position. The simulation nurse may prompt team if failure to recognize.

After stabilization, patient will ask team, “How is my baby,” and that should prompt team to assess fetal heart rate using point of care ultrasound. If asked, the simulation nurse can report that heart rate with fetal doppler is within normal limits at 140.

The learners must recognize limited resources of current center and call for transfer to level one trauma center. Case ends once report is given to receiving center.

**Critical actions:**

1. Perform primary survey with recognition of hypotension and neurovascular assessment of extremity with obvious fracture
2. Connect patient to cardiopulmonary monitors and establish two points of large bore IV access
3. Identify gravid abdomen on secondary survey and recognize the impacts of maternal physiology
4. Recognize presentation of hemorrhagic shock and start balanced blood product resuscitation considering adjuncts of TXA and calcium administration
5. Identify displaced pelvic fracture and apply pelvic binder
6. Relieve uterocaval compression to optimize maternal hemodynamics
7. Obtain fetal heart tones after stabilization
8. Arrange transport to receiving trauma center

**Case Title:** Critical Care Transport: Blunt Polytrauma in Pregnancy

**Chief Complaint:** A 24-year-old female that fell down a flight of stairs with obvious fracture of lower extremity. She is 30 weeks pregnant; this is her first pregnancy, and it has been uncomplicated with routine prenatal care.

**General Appearance:** Awake and alert, pale, obviously pregnant

**Primary Survey:**

- **Airway:** crying in pain.
- **Breathing:** tachypneic without accessory muscle use, bilateral breath sounds.
- **Circulation:** cool extremities, weak tachycardic peripheral pulse, no active extremity hemorrhage.
- **Disability:** Glasgow Coma Score 15, no obvious head trauma.
- **Exposure**: Obviously deformed lower left extremity.

**History:**

- **History of present illness:** A 24-year-old female fell down a single flight of stairs. Approximately 30 weeks pregnant, this is her first pregnancy, and it has been uncomplicated with routine prenatal care. Patient denies any loss of consciousness. Open fracture of left lower extremity. Transport by basic EMS unit, no interventions enroute.
- **Past medical history:** First pregnancy (30 weeks) uncomplicated. History of anemia during entire pregnancy.
- **Past surgical history:** None
- **Patients’ medications:** Pre-natal vitamin
- **Allergies:** None
- **Social history:** Lives at home with family, denies alcohol, tobacco, and/or illicit drug use
- **Family history:** Reviewed and non-contributory

**Secondary Survey/Physical Examination:**

- **General appearance:** Appears in pain and anxious.
- **HEENT:**
  ○ **Head:** within normal limits
  ○ **Eyes:** within normal limits
  ○ **Ears:** within normal limits
  ○ **Nose:** within normal limits
  ○ **Throat:** within normal limits
- **Neck:** within normal limits
- **Heart:** sinus tachycardia, regular, no murmurs.
- **Lungs:** Breath sounds are diminished bilaterally. No focal findings.
- **Abdominal/GI:** Gravid abdomen, fundus palpable midway between xiphoid process and umbilicus, non-tender, no bruising.
- **Genitourinary:** within normal limits
- **Rectal:** within normal limits
- **Extremities:** Left lower extremity with obvious deformity of tibia/fibula with associated laceration and bone protruding, 2+ posterior tibial and dorsalis pedis pulses, no bleeding. Tenderness to palpation at pelvis, but stable to rock and compression.
- **Back:** within normal limits
- **Neuro:** within normal limits
- **Skin:** Bruising over right chest wall.
- **Lymph:** within normal limits
- **Psych:** within normal limits

*Pelvic Radiograph*

Nondisplaced pelvic fracture.

Author’s own image

*Chest Radiograph (CXR)*

Brims, Fraser. CXR Case 044. In: Life in the Fastlane. https://litfl.com/cxr-case-044/. Published November 3, 2020. Accessed July 9, 2024. CC BY-SA 3.0

*Tib/Fib Radiograph*

Shailaja, M. Open tibia and Fibula fracture. In: Radiopedia [web].

https://radiopaedia.org/cases/open-tibia-and-fibula-fracture Published November 25, 2016. Accessed July 9, 2024. CC BY-SA 3.0

## OPERATOR MATERIALS

**Diagnosis:**

Blunt polytrauma in pregnancy with unstable pelvic fracture complicated by hemorrhagic shock and open lower extremity fracture.

**Disposition:**

Patient is transferred to a Level 1 trauma center.

## DEBRIEFING AND EVALUATION PEARLS

### Critical Care Transport: Blunt Polytrauma in Pregnancy

Trauma evaluation starts with the fundamentals of Advanced Trauma Life Support (ATLS) and the primary survey: a rapid, but methodical assessment of airway, breathing, and circulation. Clinicians must understand how the physiology of pregnancy influences resuscitation and differs from the heuristics of their typical clinical acumen.

Airway

- There is an increased risk of aspiration secondary to an increase in gastric acid production and decrease in lower esophageal sphincter tone with displacement of intra-abdominal organs by the uterus. 4,5
- There are increased Mallampati scores secondary to increased mucosal edema and friability and some patients having relative swelling of the tongue. During intubation, providers should consider video laryngoscopy for optimized view and have smaller endotracheal tubes available. 4,5

Breathing

- Oxygen consumption will increase due to increased metabolic demand of mother and fetus which decreases maternal oxygen reserve.^4^,^5^
- 2,3- diphosphoglycerate levels (DPG) will increase to allow improved oxygen-delivery capacity to the fetus, but in turn will decrease maternal oxygen reserve. 4,5
- The gravid uterus will elevate the diaphragm 3–5 cm and limit excursion, decreasing the functional residual capacity (FRC).^4^,^5^
- Estrogen and progesterone will increase respiratory rate and tidal volume. In effect, there is a baseline respiratory alkalosis pH 7.4–7.45 with a typical maternal PaCO2 of 25–32. The benefit of increased minute ventilation is an increase in oxygen delivery. The danger is that normal pH, PaO2, and PaCO2 may reflect decompensation. 4,5

Circulation

- Maternal total circulating volume will increase with significant expansion of plasma volume (1200–1600 mL) by the 3rd trimester. 4,5
- Heart rate will increase approximately 15–20 beats per minute and result in an increase in cardiac output, estimated 30–50% by 3rd trimester. 4,5
- The gravid uterus will compress the inferior vena cava (IVC) and can decrease venous return and cardiac output by up to 30%. Manual displacement of the uterus left or positioning in the left lateral tilt position are key strategies to mitigate IVC compression. 4,5
- With increased physiologic reserve, clinicians must be wary that manifestations of hemorrhagic shock can be delayed. Management should focus on hemorrhage control and balanced blood product resuscitation with Type O-negative blood until type-specific is available. 4,5

Hemorrhagic Shock

- Blood loss is the most common form of shock, a state of hypoperfusion and tissue hypoxia, in trauma patients.^6^
- The life-threatening sources of hemorrhage are the chest, abdomen, retroperitoneum, extremities, and external hemorrhage; the pillar of treatment includes hemorrhage control and restoration of intravascular volume.^6^
- Presenting features are variable based on severity, and diagnosis requires synthesis of clinical evaluation^6^:
  ○ Central perfusion: depressed mental status, thready or non-palpable central pulses
  ○ Peripheral perfusion: cool to touch, delayed capillary refill, mottling
  ○ Hemodynamics: tachycardia, narrowed pulse pressure, elevated shock index, hypotension
  ○ Positive FAST exam
- Mechanism of injury and exam suggests blood product transfusion should follow a balanced strategy, typically 1:1:1 of packed red blood cells, fresh frozen plasma, and platelets.^6^

FAST

- The goal of the exam is to identify intraperitoneal or pericardial free fluid as a source of hemorrhage or hypotension in the trauma patient.^7^
- There are four requisite views:
  ○ Right upper quadrant, looking at the hepatorenal recess (Morrison’s pouch)
  ○ Left upper quadrant, looking at the splenorenal recess
  ○ Bladder, looking at the space posterior and the peritoneum in males and rectovaginal pouch (pouch of Douglas) in females
  ○ Pericardium, looking for fluid around heart.
- The FAST exam’s high specificity for identification of intraperitoneal bleeding or pericardial fluid has made it a standard part of the evaluation of hemodynamically unstable patients with blunt traumatic injury.^7^
- The FAST exam has been studied to a limited extent in pregnancy. Retrospective studies with small sample sizes and large margins of error have shown variable sensitivity 61–83%, with consistent high specificity 94.4 – 100%. 8,9,10

Pelvic Fractures

- The pelvic ring consists of the sacrum and bilateral innominate bones (ilium, ischium, pubis); it is stabilized by the sacroiliac, sacrospinous, and sacrotuberous ligaments.
- Unstable fractures result in disruption of the pelvic ring and include anteroposterior (AP) compression, lateral compression, and vertical shear.^11^
- In this case, the patient had an AP compression fracture. The mechanism of injury involves AP forces causing external rotation at the hemipelvis and failure of the pubic symphysis resulting in diastasis and the classic “open book” teaching.^11^
- Instability and displacement can result in disruption of the pelvic venous plexus and significant hemorrhage. ATLS guidelines recommend placement of an external pelvic binder at the level of the greater trochanter to provide internal rotation of the lower extremities, temporary fixation of the pelvis, and reduction of pelvic volume to encourage tamponade and reduce hemorrhage.^6^

Fetal heart rate monitoring

- Guidelines recommend cardiotocographic monitoring for at least six hours.^12^,^13^
- In emergency transport, continuous monitoring is not a reality. Fetal heart rate should still be obtained using ultrasound.
- The measurement is performed by locating the fetal heart and centering it on your screen to apply M-mode. The user should visualize a faint sinusoidal wave that represents the fetal heartbeat. Measuring from peak-to-peak or valley-to-valley and plugging into the ultrasounds software calculator will read out your fetal heart rate.^12^
- Fetal distress with an abnormal heart rate < 110 bpm (bradycardia) or >160 bpm (tachycardia) can be the first marker of maternal compromise. 12,13

Diagnostic imaging

- The American College of Obstetricians and Gynecologists has put forth recommendations regarding diagnostic imaging in pregnancy. As it stands: CT has no absolute contraindications when clinically indicated but should have shared decision making of risks and benefits. With “few exceptions… radiography, CT scan, or nuclear medicine imaging techniques is at a dose much lower than associated with fetal harm.”^8^
- That said, radiation is a teratogen and can be lethal to the embryo. During weeks two to eight, there is risk for congenital anomalies and growth restriction. During weeks eight to fifteen, the nervous system is most susceptible and can cause intellectual disability and microcephaly.^14^

## SIMULATION ASSESSMENT

*Critical Care Transport: Blunt Polytrauma in Pregnancy*

Learner: _________________________________________

***Assessment Timeline***

This timeline is to help observers assess their learners. It allows observer to make notes on when learners performed various tasks, which can help guide debriefing discussion.

**Critical Actions:**

□ Perform primary survey with recognition of hypotension and neurovascular assessment of extremity with obvious fracture
□ Connect patient to cardiopulmonary monitors and establish two points of large bore IV access
□ Identify gravid abdomen on secondary survey and recognize the impacts of maternal physiology
□ Recognize presentation of hemorrhagic shock and start balanced blood product resuscitation considering adjuncts of TXA and calcium administration
□ Identify displaced pelvic fracture and apply pelvic binder
□ Relieve uterocaval compression to optimize maternal hemodynamics
□ Obtain fetal heart tones after stabilization
□ Arrange transport to receiving trauma center

**Summative and formative comments:**

**Milestones assessment:**
