# Supplementary figures and images for: Critical Care Transport: Blunt Polytrauma in Pregnancy
Source: J Educ Teach Emerg Med. 2025 Jul 31;10(3):S1–S24. doi: 10.21980/J81366 (PMC12320994; doi:10.21980/J81366)

## Slide 1
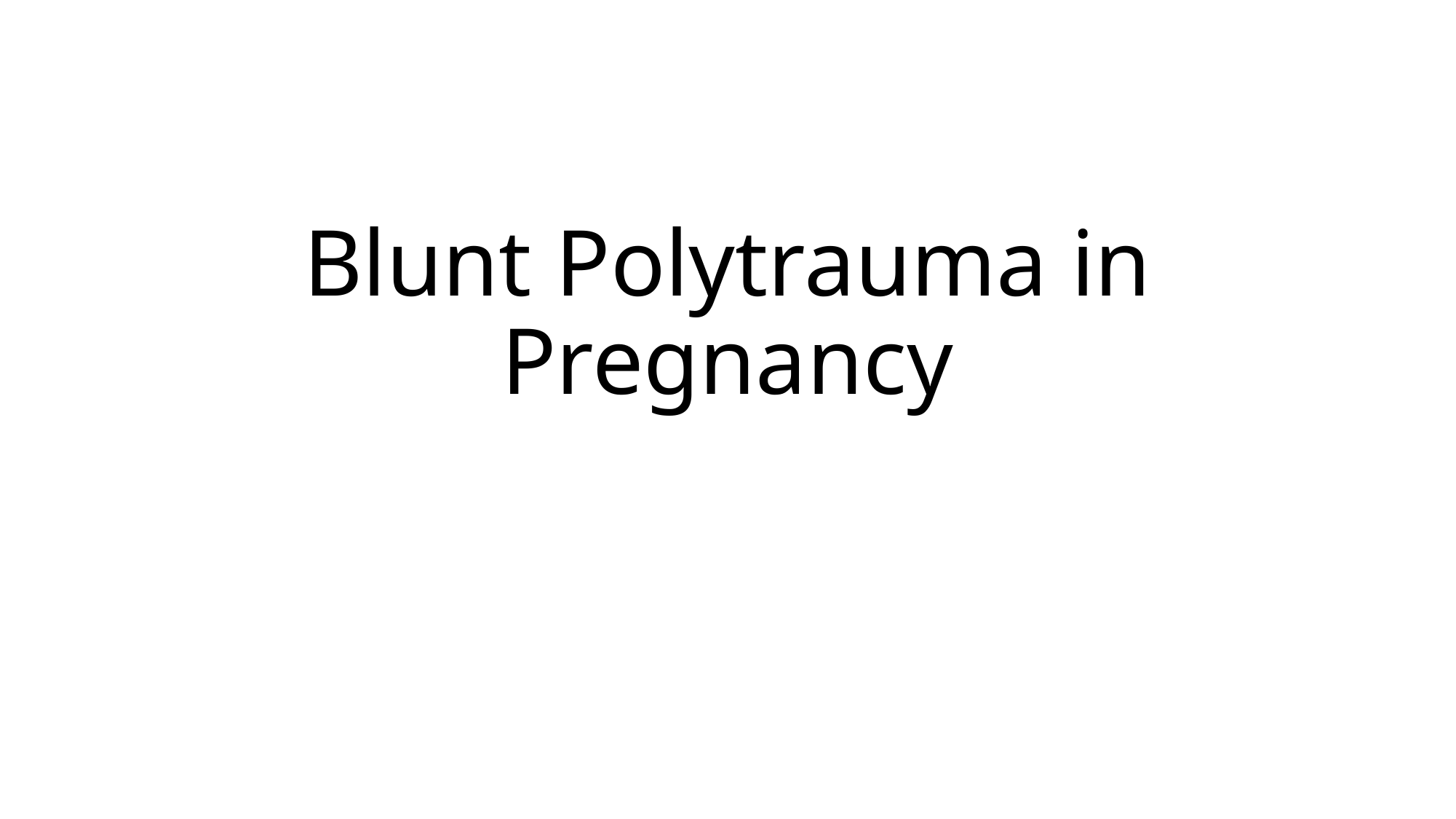

# Blunt Polytrauma in Pregnancy

## Slide 2
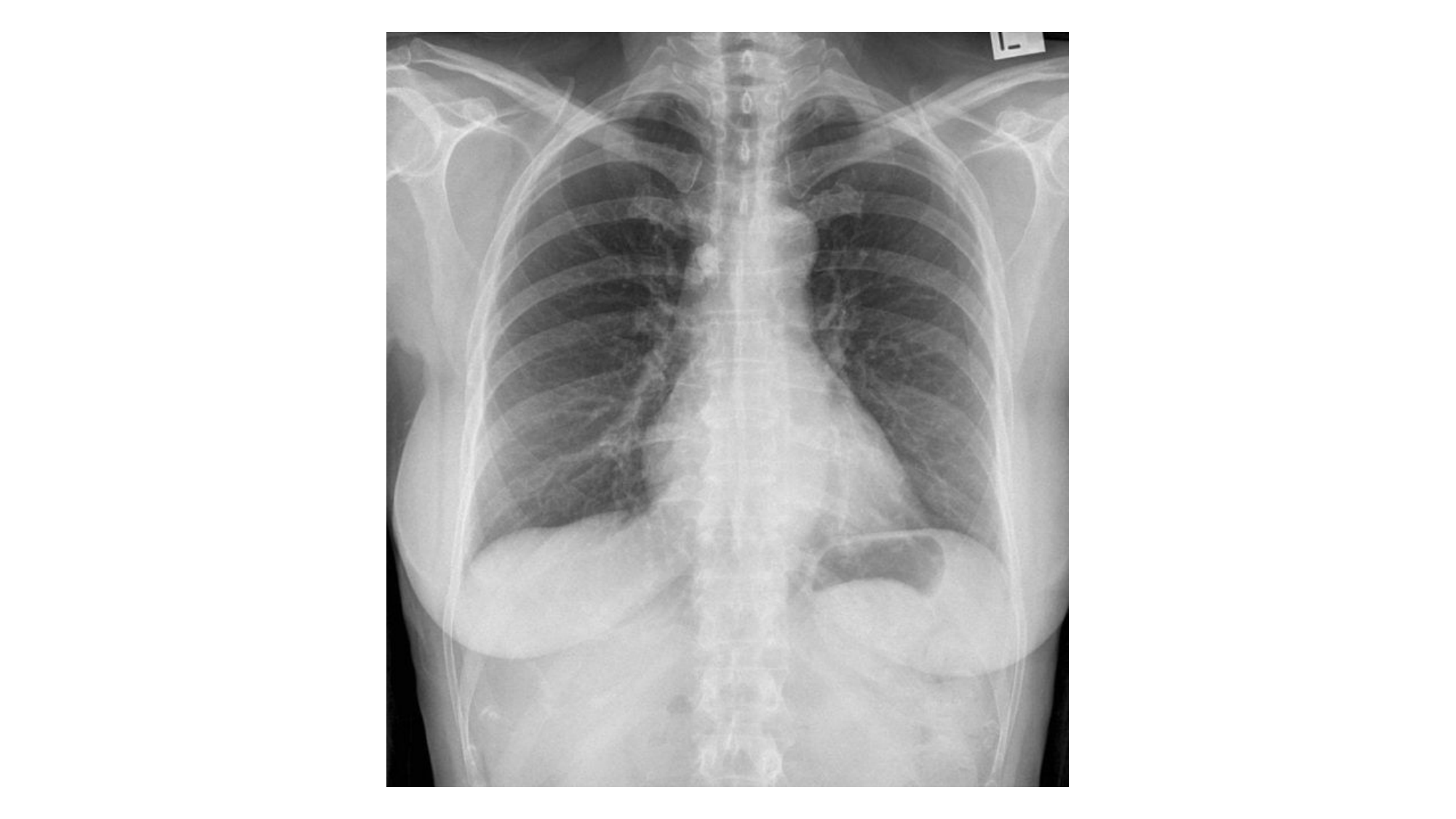

## Slide 3
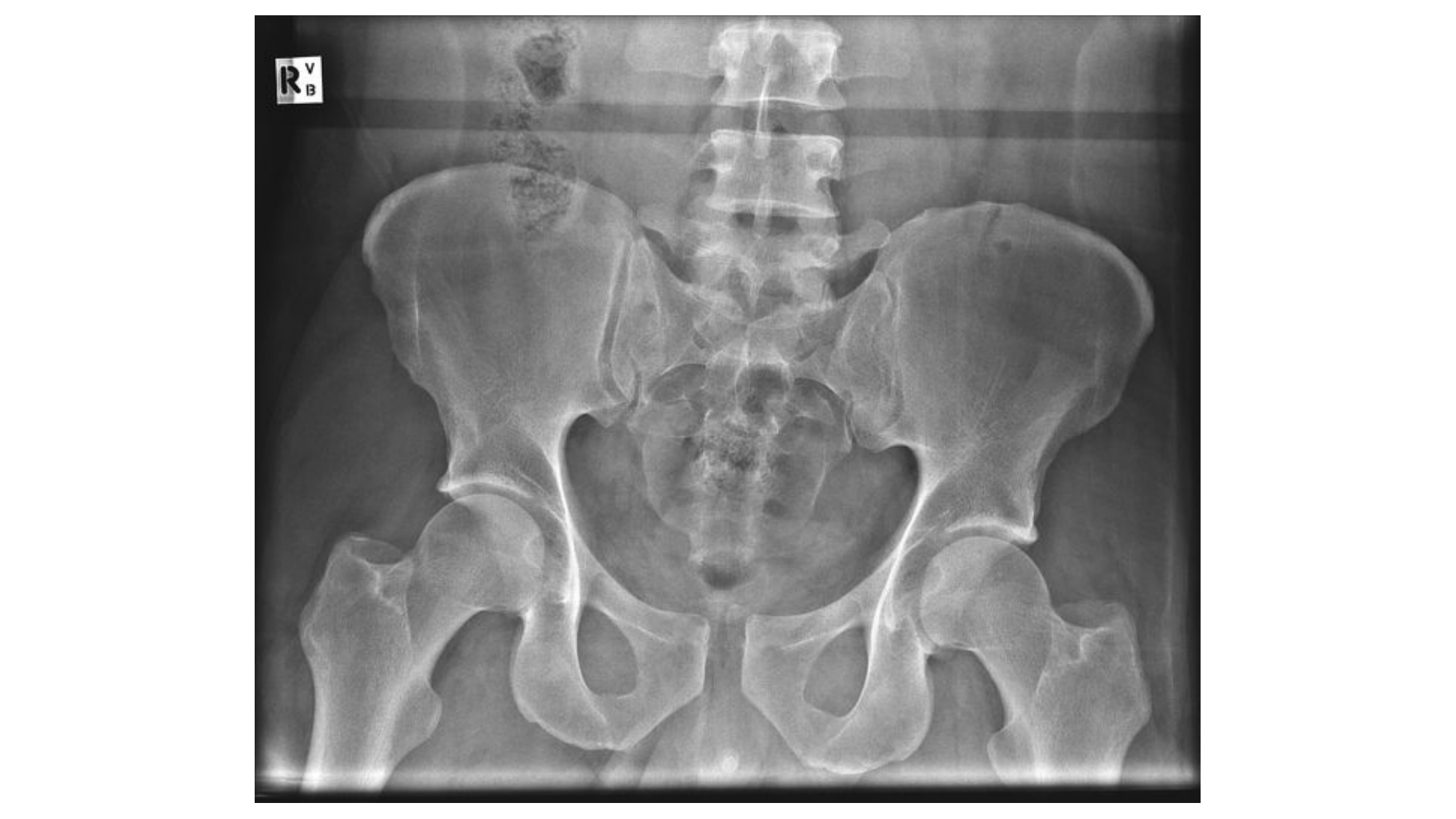

## Slide 4
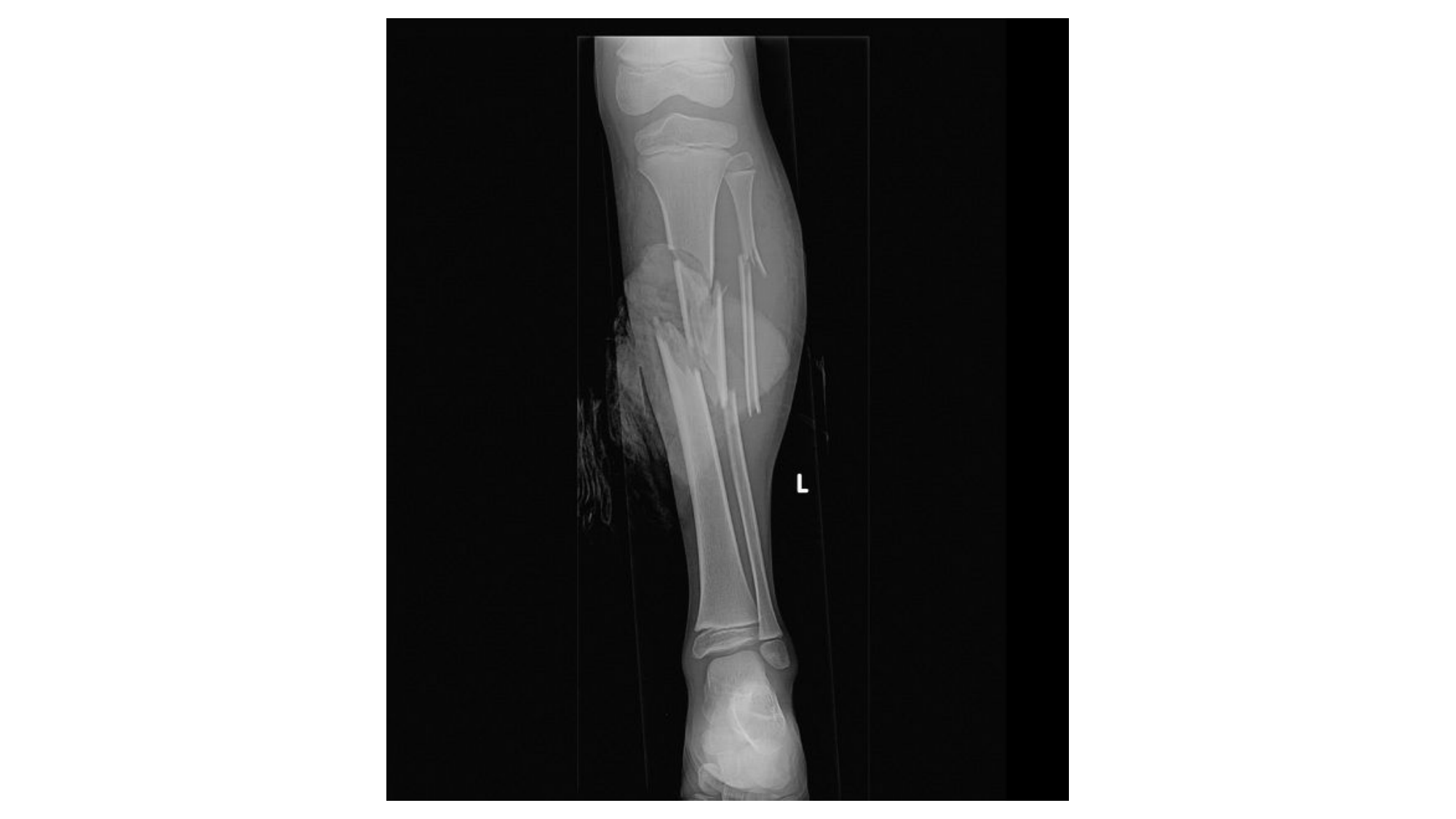

Supplement: Supplementary file 1 [file 10-3-S1-Supp1.pptx]
